# Assessment of Risk Factors for Delayed Colonic Post-Polypectomy Hemorrhage: A Study of 15553 Polypectomies from 2005 to 2013

**DOI:** 10.1371/journal.pone.0108290

**Published:** 2014-10-01

**Authors:** Qiang Zhang, Sheng li An, Zhen yu Chen, Feng-Hua Fu, Bo Jiang, Fa chao Zhi, Yang Bai, Wei Gong

**Affiliations:** 1 Department of Gastroenterology, Nanfang Hospital, Southen Medical University, Guangzhou, China; 2 Department of Bio-Statistics, School of Public Health and Tropical Medicine, Southern Medical University, Guangzhou, China; University Hospital Llandough, United Kingdom

## Abstract

**Background and Aim:**

Delayed colonic postpolypectomy bleeding is the commonest serious complication after polypectomy. This study aimed to utilize massive sampling data of polypectomy to analyze risk factors for delayed postpolypectomy bleeding.

**Patients and Methods:**

The endoscopic data of 5600 patients with 15553 polyps removed (2005 to 2013) were analyzed retrospectively through univariate analysis and multiple logistic regression analysis to evaluate the risk factors for delayed bleeding.

**Results:**

Delayed postpolypectomy bleeding occurred in 99 polyps (0.6%). The rates of bleeding for different polypectomy methods including hot biopsy forcep, biopsy forcep, Argon Plasma Coagulation (APC), Endoscopy piecemeal mucosal resection (EPMR), Endoscopic Mucosal Resection (EMR), and snare polypectomy were 0.1%, 0.0%, 0.0%, 6.9%, 0.9% and 1.0%, respectively. The risk factors for delayed bleeding were the size of polyps over 10 mm (odds ratio [OR] = 4.6, 95% CI, 2.9–7.2), pathology of colonic polyps (inflammatory/hyperplastic, OR = 1; adenomatous, OR = 1.4, 95% CI, 0.7–2.6; serrated, OR = 1.5, 95% CI, 0.2–11.9; juvenile, OR = 4.3, 95% CI, 1.8–11.0; Peutz-Jegher, OR = 3.3, 95% CI, 1.0–10.7), and immediate postpolypectomy bleeding (OR = 2.9, 95% CI, 1.4–5.9). In addition, although polypectomy method was not a risk factor, compared with hot biopsy forcep, snare polypectomy, EMR, and EPMR had increased risks of delayed bleeding, with ORs of 3.2 (0.4–23.3), 2.8 (0.4–21.7) and 5.1 (0.5–47.7), respectively.

**Conclusion:**

Polyp size over 10 mm, pathology of colonic polyps (especially juvenile, Peutz-Jegher), and immediate postpolypectomy bleeding were significant risk factors for delayed postpolypectomy bleeding.

## Introduction

Hemorrhage is a relatively common complication when performing colonic polypectomy [1], [2]. Polypectomy bleeding includes immediate and delayed bleeding [3]. A multicenter study found the incidence of immediate bleeding during polypectomy to be 2.8% [4], while the incidence of delayed bleeding was 0.3–0.6% [2], [5], [6]. Although the incidence of immediate bleeding is relatively high, it is easier to immediately stop the bleeding by effective methods, such as endoclips [7]–[9], cauterization and adrenaline injection [10]. However, it is difficult to predict the timings of delayed hemorrhage. Some research had shown that delayed bleeding occurred in 3–7 days [11]–[13], while others found that it occurred in 2–14 days [2]. There was a single case of delayed bleeding after 29 days following polypectomy [3]. Delayed post-polypectomy hemorrhage is difficult to detect initially, and it becomes more difficult to perform emergency diagnostic and therapeutic endoscopic treatment once bleeding has occurred. Thus it is imperative to identify the risks of delayed polypectomy bleeding in advance to avoid serious complications resulting from delayed hemorrhage in clinic. At present, the size of the polyp [2], [4], [14]–[16], histological classification [14]–[17], right hemi-colon polyps [18], [19], [21], recent usage of anticoagulants [5], [20], and hypertension [2] are risk factors for delayed hemorrhage. Because the incidence of delayed polypectomy bleeding is low, the sample sizes of patients with hemorrhage included in previous clinical studies were small, so it is necessary to mine relative risk factors from large sample size. In this study, we aimed to utilize massive sampling data of patients who underwent polypectomy to analyze the risk factors for delayed postpolypectomy bleeding.

## Methods

### Patients and methods

Our research subjects were patients treated with colonic polypectomy from January 2005 to June 2013 in the Endoscopy Center of Nanfang Hospital, Guangzhou, China, which is recognized as the “National Key Discipline” which is afforded special recognition and support from the Chinese government for conducting important research. All treated patients were hospitalized and examined thoroughly, and some informations such as their blood pressure and coagulation functions were evaluated. After a full evaluation of the clinical indications, they were then treated with polypectomy. Relevant polypectomy contraindications are as follows: serious heart and lung diseases, unable to receive endoscopic therapy; coagulation dysfunction and the tendency of hemorrhage; too large polyp base, greater than 1.5 cm; the polypoid carcinoma having been infiltrated and deteriorated. Some patients possessed multiple polyps, and most of them had their polyps removed by a single endoscopic treatment. All patients included in this study provided written informed consent for the treatment. This retrospective study had been approved by Ethical Committee of Nanfang Hospital of Southern Medical University. All data had been anonymized and deidentified.

The group of bleeding included cases with delayed postpolypectomy bleeding. Delayed postpolypectomy hemorrhage refers to the hemorrhage occurring after the completion of polypectomy and after the patient has been sent back to the ward. The criteria of delayed bleeding included one of two conditions: 1. No bleeding during the polypectomy, but with bloody stool being detected after the polypectomy; 2. Bleeding during the polypectomy and successful hemostasis, but with continuous bloody stool after polypectomy. The cases of delayed bleeding were firstly managed conservatively by supportive care, and we performed second colonoscopy for those cases of continuing bleeding or haemodynamic compromise not able to be managed with supportive care. The control group included patients without delayed bleeding during hospitalization. During hospitalization, the control group had no sustained bloody stool and their vital signs remained stable, as did their hemoglobin levels. After polypectomy, the period of hospitalization varied according to the patients' condition. Generally, the hospital stay was 2∼6 days.

### Polypectomy

Different polypectomy methods were applied based upon the size and morphology of polyps. Argon plasma coagulation (APC), hot biopsy forceps and biopsy forceps were used to remove smaller polyps, while snare polypectomy and endoscopic mucosal resection (EMR) were used for larger polyps. Snare polypectomys were used to resect pedunculated or subpedunculated polyps, sessile polyps were generally removed by EMR, and the flat polyps too wide to completely remove at once were resected by endoscopic piecemeal mucosal resection (EPMR). If bleeding occurred during polypectomy, endoclip, hot biopsy forceps or adrenaline injection were applied to control the bleeding.

### Data acquisition and explanation

The data we collected included the age and gender of patients, the numbers of accumulated colonoscopy cases, including polypectomy cases of an endoscopic doctor. The accumulated number of cases indicated total cases performed by the doctors before the day of polypectomy. The number of colonoscopy cases represented their colonoscopy and polypectomy experience.

Data regarding polyps was collected with the following parameters: the location of the polyps, polyp size, histological classification, atypia and method of polypectomy. Polyp location included the left hemi-colon (descending colon, sigmoid colon and rectum) and right hemi-colon (appendix, ascending colon and transverse colon). Simultaneously, the appendix, hepatic flexure and splenic flexure were also recorded as individual parts of the colon. Histology was either inflammatory, hyperplastic, adenomatous, serrated, juvenile, or Peutz-Jeghers polyp. Atypia was classified as mild, moderate, severe and carcinogenic and methods of polypectomy included snare polypectomy, EMR, EPMR, APC, biopsy forceps and hot biopsy forceps. The occurrence of immediate postpolypectomy bleeding and the number of resected polyps in each patient were also recorded. Moreover, To clarify, the use of biopsy forceps inevitably caused temporary bleeding during polypectomy, and such bleeding would not be defined as immediate postpolypectomy bleeding.

### Statistical analysis

All the statistical analyses were performed using SPSS, version 20 (SPSS, Chicago, IL, USA). For normally distributed data, Student t test was used for continuous variables and chi square test was used for categorical variables. Mann-Whitney U test was used for data that are non-normally distributed. First of all, univariate analyses were performed for all possible risk factors. Identified significant variables were taken as potential risk factors and were included in the multivariate logistic regression model. Odds ratio (OR) and 95% confidence interval (CI) are reported for significant variables revealed by the multivariate analysis. A P-value (two-side) of less than 0.05 was considered statistically significant.

## Results

### Incidence of post-polypectomy hemorrhage and the interval between polypectomy and hemorrhage

**Figure 1:**
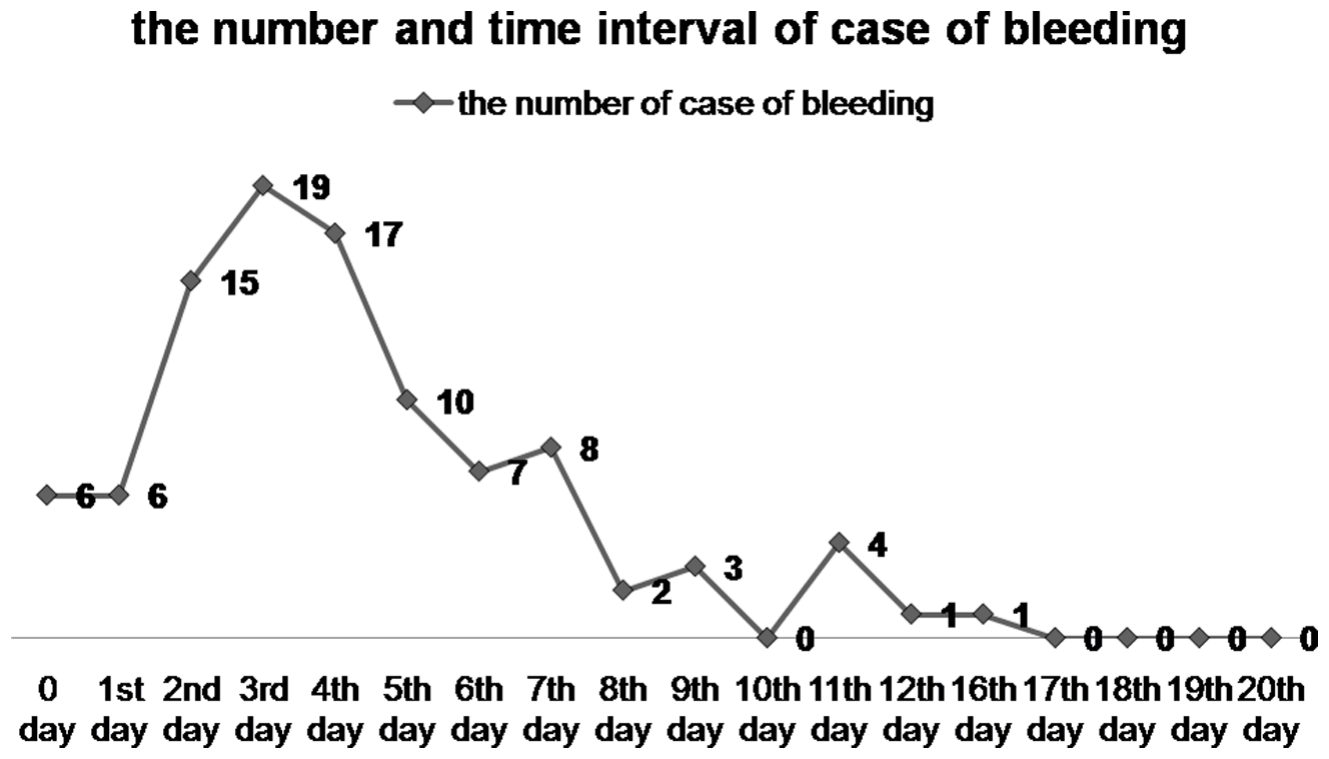
The number of cases of delayed bleeding, and time interval between the time of polypectomy and the occurrence of delayed hemorrhage. 0 day, 1st day, 2nd day, et al represent the time intervals.

As shown in Table 1, a total of 15553 polyps were resected in 5600 patients and the incidence of hemorrhage was 0.6% (99/15553). The mean size of polyps removed by snare polypectomy was 11.0±8.8 mm and the incidence of hemorrhage was 1.0% (76/7937). The mean size of polyps removed by endoscopic mucosal resection (EMR) was 10.0±8.0 mm and the incidence of hemorrhage was 0.9% (15/1596). The mean size of polyps removed by piecemeal EMR (EPMR) was 33.0±21.0 mm and the incidence of hemorrhage was 6.9% (6/87). The mean size of polyps removed by argon plasma coagulation (APC) was 5.0±2.0 mm and the incidence of hemorrhage was less than 0.1% (1/3155). The mean size of polyps removed by biopsy forceps was 3.0±1.0 mm and the incidence of hemorrhage was 0 (0/2054). The mean size of polyps removed by hot biopsy forceps was 4.0±2.0 mm and the incidence of hemorrhage was 0.1% (1/724). Delayed hemorrhage occurred in 99 cases of colonic polypectomy, of which 6 (6.0%) cases had delayed hemorrhage on the day of polypectomy and 1 case (1.0%) had delayed hemorrhage up to 16 days after the polypectomy. The interval between the time of polypectomy and the occurrence of delayed hemorrhage was 4.0±2.9 days with 93% of the hemorrhage occurring during this period of time (Figure 1).

**Table 1:** The average sizes of polyps dealt with different endoscopic methods and the rates of post-polypectomy hemorrhage of each method to handle polyps.

Endoscopic method	Polyp size(mm)#	Incidence(%)
**All therapeutic endoscopies**	8.0(7.9)	99/15553(0.6)
**Snare polypectomy**	11.0(8.8)	76/7937(1.0)
**EMR**	10.0(8.0)	15/1596(0.9)
**EPMR**	33.0(21.0)	6/87(6.9)
**APC**	5.0(2.0)	1/3155(0.0)
**Biopsy forcep**	3.0(1)	0/2054(0.0)
**Hot biopsy forcep**	4.0(2.0)	1/724(0.1)

# , mean (standard deviation).

### Risk factors for delayed colonic postpolypectomy hemorrhage

The results of per-polyp univariate analysis showed that polyp size, polyp pathology, atypia, the resection methods of polyps, and immediate postpolypectomy bleeding were potential risk factors for delayed postpolypectomy hemorrhage (Table 2). As shown in Table 3, multivariate logistic regression analysis indicated that polyp pathology was a significant risk factor (P = 0.008). The odds ratio (OR) of the inflammatory/hyperplastic polyps was referenced at 1, and the ORs of the other histologic types were as follows: adenomatous polyps 1.4 (95% CI 0.7–2.6, *p* = 0.320), serrated polyps 1.5 (95% CI 0.2–11.9, *p* = 0.690), juvenile polyps 4.3 (95% CI 1.8–11.0, *p* = 0.001), and Peutz-Jeghers (P-J) polyps 3.3 (95% CI 1.0–10.7, *p* = 0.040). The other significant factors were polyps larger than 10 mm (OR = 4.6, 95% CI 2.9–7.2, *p*<0.001), and immediate postpolypectomy bleeding (OR = 2.9, 95% CI 1.4–5.9, *p* = 0.004). In addition, Although polypectomy method was not a risk factor, compared with hot biopsy forcep, biopsy forcep and APC, snare polypectomy, EMR, and EPMR had relatively high OR values of 3.2 (0.4–23.3), 2.8 (0.4–21.7) and 5.1 (0.5–47.7), respectively.

**Table 2:** Univariate analysis of risk factors for delayed colonic postpolypectomy hemorrhage.

	non- hemorrhagic group	hemorrhagic group	P Value (2-sided)
**Endoscopic experience** #	2922(5000)	2466(3505)	0.133
**Polyp location 1 (%)**			0.30
Left hemi-colon	11010/14819 (74.3)	78/99 (78.8)	
Right hemi-colon	3809/14819 (25.7)	21/99 (21.2)	
**Polyp location 2 (%)**			0.90
Appendix	689/14880 (4.6)	3/99 (3.0)	
Hepatic flexure	309/14880 (2.1)	2/99 (2.0)	
Splenic flexure	61/14880 (0.4)	0/99 (0.0)	
Rectum	3971/14880 (26.7)	28/99 (28.3)	
Colon	9850/14880 (66.2)	66/99 (66.7)	
**Polyp size** 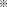	8.8(7.8)	19.0(13.2)	0.000
**Polyp pathology (%)**			0.000
Inflammatory/hyperplastic	4792/12942(37.0)	12/92(13.0)	
Adenomatous	7323/12942 (56.6)	66/92 (71.7)	
Serrated	338/12942 (2.6)	1/92 (1.1)	
Juvenile	218/12942 (1.7)	9/92 (9.8)	
Peutz-Jehger	271/12942 (2.1)	4/92 (4.3)	
**Atypia (%)**			0.000
Mild	4484/13748 (32.6)	27/91 (29.7)	
Moderate	1856/13748 (13.5)	19/91 (20.9)	
Severe	798/13748 (5.8)	15/91 (16.5)	
Canceration	120/13748 (0.9)	3/91 (3.3)	
No atypia	6490/13748 (47.2)	27/91 (29.7)	
**Polypectomy methods (%)**			0.000
Snare polypectomy	7431/14794(50.2)	76/99(76.8)	
EMR	1542/14794 (10.4)	15/99 (15.2)	
EPMR	81/14794 (0.5)	6/99 (6.1)	
APC	2989/14794 (20.2)	1/99 (1.0)	
Biopsy forcep	2049/14794 (13.9)	0/99 (0.0)	
Hot biopsy forcep	702/14794 (4.7)	1/99 (1.0)	
**Immediate postpolypectomy bleeding (%)**	256/15467(1.7)	11/99 (11.1)	0.000

# , median (interquartile range);

^
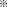
^, mean (standard deviation).

**Table 3:** Multivariate analysis of risk factors for delayed colonic postpolypectomy hemorrhage.

	Odds Ratio	95% Confidence Interval	P Value
**Polypectomy methods**			0.790 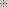
Hot biopsy forcep	1		
Snare polypectomy	3.2	0.4–23.3	0.250
EMR	2.8	0.4–21.7	0.320
EPMR	5.1	0.5–47.7	0.150
APC	0	0	0.990
Biopsy forcep	0	0	0.990
**Polyp size (mm)**			<0.001
≤10	1		
>10	4.6	2.9–7.2	
**Polyp pathology**			0.008
Inflammatory/hyperplastic	1		
Adenomatous	1.4	0.7–2.6	0.320
Serrated	1.5	0.2–11.9	0.690
Juvenile	4.3	1.8–11.0	0.001
Peutz-Jehger	3.3	1.0–10.7	0.040
**Immediate postpolypectomy bleeding**			0.004
No	1		
Yes	2.9	1.4–5.9	

^
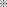
^, Polypectomy methods were not risk factors, with p>0.1, but the polypectomy methods were still selected to enter the logistic regression model.

Moreover, some patients have multiple colonic polyps, and multiple polyps are usually resected all at once. Our result showed that the number of resected polyps does not seem to be a risk factor of delayed bleeding (Table 4 and 5).

**Table 4:** Univariate analysis of age, gender and number of resected polyps for delayed colonic postpolypectomy hemorrhage based on patients.

	Non- hemorrhagic patients (n = 5507)	Hemorrhagic patients (n = 93)	P-value
**Age (Mean±SD)**	53±14	47±16	0.001 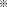
**Male(%)**	3874(70.3)	70(75.3)	0.208#
**Number of resected polyps** & **(Median(P_25_–P_75_))**	1(1–3)	1(1–3)	0.148§

^
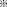
^, independent t test;

# , Pearson χ^2^;

§ , Mann Whitney test;

& , some patients have multiple colonic polyps, and these polyps are usually resected all at once. Number of resected polyps is represented in Median(P_25_–P_75_).

**Table 5:** Univariate analysis of number of resected polyps for delayed colonic postpolypectomy hemorrhage based on patients and different polypolypectomy methods.

	Non- hemorrhagic patients N&/(Median(P_25_–P_75_)	Hemorrhagic patients N&/(Median(P_25_–P_75_)	P-value§
**APC**	407/3(1–8)	1/60 (-)	-
**EMR**	1170/1(1–2)	14/1(1–2.75)	0.509
**EPMR**	80/1(1–1)	6/1(1–1.75)	0.062
**Biopsy forcep**	1388/1(1–2)	0/-	-
**Hot biopsy forcep**	377/1(1–2.5)	1/1(-)	-
**Snare polypectomy**	4647/1(1–2)	71/1(1–3)	0.944

§ , Mann Whitney test;

& , “N” refers to the number of patients with colonic polyps resected by a certain polypectomy method.

## Discussion

In this study, we analyzed the risk factors for delayed postpolypectomy hemorrhage. In addition to polyp size having been reported as a risk factor, we further identified that polyp pathology, especially juvenile polyps and P-J polyps, immediate postpolypectomy bleeding were significant risk factors for delayed postpolypectomy bleeding. Among endoscopic methods for polypectomy, EPMR had a highest risk of delayed bleeding.

Polyp size is generally recognized as a risk factor for delayed postpolypectomy bleeding [2], [4], [14]–[16]. Our study showed that polyp pathology was also one of the risk factors for delayed bleeding. In our study polyp pathology was further classified into inflammatory/hyperplastic, adenomatous, serrated, juvenile, and P-J polyps. The delayed bleeding rate for inflammatory polyps and hyperplastic polyps (4792 cases) was 0.2%. Compared with inflammatory/hyperplastic polyps (OR value defined as 1), there was not an increased risk of hemorrhage risk for adenomatous polyp (OR = 1.8). Both juvenile polyp and Peutz-Jegher belong to hamartomatous polyp. Usually, the resected juvenile polyp and Peutz-Jegher have a relatively large polyp size. Thus, the two pathological features and size of the polyp are two factors that are correlated to some extent. However, through multivariate analysis, it is found that both juvenile polyp and Peutz-Jegher are risk factors contributing to delayed hemorrhage, and their hemorrhage risks were significantly higher with odds ratios of 5.7 and 4.3 than inflammatory/hyperplastic polyp. Two other studies also found that polyp pathology is a risk factor, however, one of the studies focused on hemorrhage occurring during polypectomy [14]. The subject of the other study was pedunculated polyps larger than 15 mm, and the polypectomy methods included use of an endoclip or endoloop [17]. A study conducted by Hirotsugu Watabe *et.al* [2] demonstrated that adenomatous polyp and adenocarcinoma were not risk factors, but the sample size of bleeding cases was relatively small (n = 37). In our study, we compared and analyzed different histologic classifications, and the effects of these classifications on delayed postpolypectomy bleeding were comprehensively evaluated.

Regarding the methods used for polypectomy, the usage of APC, biopsy forcep and hot biopsy forcep to treat smaller polyps had very low percentage rates of delayed hemorrhage (0.0%, 0.0% and 0.1%, respectively). The use of snare polypectomy, EMR and EPMR resulted in higher bleeding probabilities (1.0%, 1.0% and 6.9%, respectively). In general, the selection of polypectomy methods depends very much on the size of polyps. This study incorporates both polypectomy method and polyp size as two factors for analysis, which might have a certain degree of mutual influence between the two factors. The final multivariate analysis shows that polypectomy method is not a risk factors for delayed postpolypectomy bleeding. In the research conducted by Hirotsugu Watabe *et.al* [2], hot biopsy forcep, snare polypectomy and EMR had no significant differences in the risk of delayed bleeding. Although polypectomy methods were not risk factors, there were significant different rates of delayed bleeding among polypectomy methods, for example, compared with APC and hot biopsy forcep, EPMR had a high risk of bleeding, so we still made polypectomy methods enter the logistic regression analysis.

In our study, immediate postpolypectomy bleeding was also a risk factor for delayed postpolypectomy hemorrhage. In the research of Hyun S, Kim *et.al* [4], the grade of immediate postpolypectomy bleeding was divided by severity, including mild, spontaneous hemorrhage and severe projectile hemorrhage. Our research did not classify the bleeding severity in such detail, nevertheless, the severity of bleeding might well result in variations in the risk of delayed postpolypectomy hemorrhage. This remains to be further investigated. Usually, multiple colonic polyps are resected all at once, and we did not found that number of resected polyps is a risk factor of delayed bleeding.

The literature had reported [18], [21] that the location of polyps in the right hemi-colon, specifically the appendix, was a risk factor for delayed postpolypectomy hemorrhage, while our research did not confirm these results. One possible reason could be that the polyp locations vary between Western and Chinese patients. There were studies in the literature showing that in Asian people, the percentage of polyps located in the left hemi-colon was 57%, while the percentage in the right hemi-colon was 30%, whereas in Western people polyps were equally distributed with 49% of polyps in each of the left and right hemicolon. In our study, 74% of colonic polyps were located in the left hemi-colon [22].

There were two key limitations in this study: 1. The retrospective study itself was limited. The sample size of our research was large enough to reduce the bias based on case selection. All polypectomy cases within a defined period were included for analysis, but during multivariate analysis, only a small part of cases with incomplete data were excluded. Compared to the large sample size, excluding cases with incomplete data could have little effect; 2. Some patient-related factors such as hypertension and anticoagulant usage had been reported as risk factors for delayed postpolypectomy hemorrhage[2], [5], [20]. Our research did not consider patient-related risk factors. Our study mainly reflected the influence of the endoscopist and polyp-related factors on delayed post-polypectomy hemorrhage. We'd like to state that the patients' conditions and co-morbid diseases were comprehensively evaluated before patients receiving polypectomy. Only those patients having not contraindications of polypectomy received polypectomy. Therefore these patient-related risk factors were controlled as far as was possible before polypectomy. Secondly, titanium clip as a prophylactic method for post-polypectomy hemorrhage after polypectomy was widely used. However, in our study cases only a small quantity of polypectomy was used with the clip, so titanium clip had not been analyzed in our study. One study by Kazuhiko Shioji *et al* suggested that employing titanium clips to close incisions after polypectomy had not the influence on the delayed hemorrhage rate [23]. Maybe, it was little influence on the result of our study that titanium clip was not considered in our study.

## Conclusions

To sum up, our study identified polyp size over 10 mm, pathology of colonic polyps (especially juvenile, Peutz-Jegher), and immediate postpolypectomy bleeding as significant factors of delayed postpolypectomy hemorrhage. Therefore, after polypectomy, clinical doctor should give special attention to these patients to avoid the serious complications resulting from delayed bleeding.
